# Comparing the Brief Assessment of Cognitive Health and Montreal Cognitive Assessment: Test‐retest reliability and sensitivity to cognitive change in older adults

**DOI:** 10.1002/alz.094295

**Published:** 2025-01-09

**Authors:** Darlene P Floden, Kelsey Curran, Olivia Hogue, Kamini Krishnan, Saket A Saxena, Claire Sonneborn, Michael B Rothberg, Anita D Misra‐Hebert, Alex P Milinovich, Elizabeth R Pfoh, Robert J Fox, Michael W Kattan, Robyn M Busch

**Affiliations:** ^1^ Cleveland Clinic, Cleveland, OH USA

## Abstract

**Background:**

Test‐retest reliability for existing cognitive screening tests is typically poor and most have ceiling effects and restricted score ranges that mask the presence of subtle decline. The Brief Assessment of Cognitive Health (BACH) is a computerized cognitive screening tool that patients complete independently. It includes a complex memory test without ceiling effects and brief mood and history questions. The BACH generates a probability score for cognitive impairment that is highly accurate at predicting impairment on neuropsychological testing. The goal of this study was to determine if the psychometric characteristics of BACH (i.e., test‐retest reliability and sensitivity to cognitive change) are superior compared to a commonly used screening test, the Montreal Cognitive Assessment (MoCA).

**Method:**

Ninety‐seven participants completed the BACH and MoCA at two timepoints. A mixed effects model was fit to derive between‐ and within‐subjects variability to calculate the intraclass correlation coefficient (ICC) to assess test‐retest reliability of the screening tools. A subset of 52 participants completed the same neuropsychological battery at both timepoints and individual composite cognitive change scores were calculated. Pearson correlations were used to determine the strength of relationships between the composite cognitive change score and change scores on the MoCA and BACH.

**Result:**

On average, the ICC sample was 68 years‐old with 15 years education, and 56% were female. The median time between test sessions was 384 days (range 246‐1211). ICC for the BACH probability of impairment score was 0.59 (moderate reliability) whereas the ICC for the MoCA was 0.48 (poor reliability). For the cognitive testing sample (average age = 72 years, 16 years ed, 54% female), median time between test sessions was 336 days (range 263‐426). Composite cognitive change score was moderately related to BACH probability change (r = ‐0.49; CI = [‐0.68, ‐0.26]) and strongly related to BACH memory score change (r = 0.55; CI = [0.32, 0.71]). Composite cognitive change score was weakly associated with MoCA change score (r = 0.12; CI = [‐0.17, 0.38]).

**Conclusion:**

The BACH demonstrated moderate to strong test‐retest reliability and sensitivity to cognitive change, while observed MoCA psychometrics were below the cutoffs recommended for clinical practice. The BACH is a more accurate tool for cognitive surveillance in older adults.

